# Mortality in offspring with parental criminal convictions: A population-based register study from Sweden

**DOI:** 10.1016/j.ssmph.2026.101928

**Published:** 2026-05-02

**Authors:** Venla Berg, Ralf Kuja-Halkola, Henrik Larsson, Paul Lichtenstein, Antti Latvala

**Affiliations:** a Population Research Institute, Väestöliitto (the Family Federation of Finland), Finland; b Department of Medical Epidemiology and Biostatistics, Karolinska Institutet, Sweden; c Institute of Criminology and Legal Policy, University of Helsinki, Finland; d School of Medical Sciences, Örebro University, Sweden

**Keywords:** Child mortality, Offending behavior, Parental incarceration, Parental criminal convictions, Cause-specific mortality, Register research, Full-population data

## Abstract

**Background:**

Parental offending behavior is associated with poorer child mental and physical health, but less in known about its association with child mortality. Further, research is focused on the United States, which is unique in its societal structure and mass incarceration.

**Methods:**

Using population-based register data on all births in Sweden in 1973–2013 (N = 4.2 million) and Cox regressions, we examined the associations of mother's and father's non-violent and violent criminal convictions with offspring all-cause and cause-specific mortality in infancy, childhood, and adolescence.

**Results:**

Parental convictions were associated with increased offspring all-cause mortality in infancy (11–39% higher mortality risk compared to children without parental convictions), ages 1–9 (11–47% higher risk), and ages 10–19 (12–78% higher risk). Parental convictions were associated with heightened risk of death due to infections and communicable diseases among infants and in ages 10–19 (44–100% higher risk), with accident mortality until age 19 (31–178% higher risk), and with suicide mortality among 10–19-year-old children (26–112 % higher risk). Mortality risks were higher among children of parents with violent convictions and among children whose mothers had convictions.

**Conclusions:**

In addition to more severe offending behavior, non-violent parental convictions were also associated with offspring mortality. Cause-specific mortality patterns indicate that the associations may involve parental as well as children's own behavior. Comprehensive and long-term measures and support are needed to prevent intergenerational adversity related to parental offending.

## Highlights

• Parental criminal convictions associated with higher offspring mortality until age 20.
• Violent and maternal convictions linked to the greatest mortality risks.
• Elevated cause-specific mortality for infections, accidents, and suicide.
• Non-violent convictions also contribute to increased mortality.

## 1 Introduction

Even though most people never engage in delinquent behavior, criminal offending is relatively prevalent, with many offenders persisting in criminal behavior well into adulthood (Jolliffe et al., 2017). Many children thus have parents with a history of offending behavior, and many are also exposed to parental criminality during their childhood (Zoutewelle-Terovan & Skardhamar, 2016). For example, in the United States, nearly eight percent of children experience paternal imprisonment by the age of 14 (Wildeman & Andersen, 2015). Parental offending potentially has profound implications for children's development, somatic and mental health, and educational outcomes (Hutson et al., 2023; Wildeman et al., 2018).

Parental incarceration, for instance, is linked to children's lower educational attainment and an increased likelihood of internalizing, externalizing, and antisocial behaviors, as well as poorer mental and somatic health during childhood and adolescence (Bomysoad & Francis, 2022; Haskins, 2015; Herreros-Fraile et al., 2023; Tolliver et al., 2024; Turney, 2022). In addition, parental incarceration near the time of childbirth is correlated with worse infant birth outcomes, partly due to the mother's compromised health behaviors and reduced access to medical care and screenings during pregnancy (Dumont et al., 2014; Lee et al., 2023; Testa et al., 2020; Yi et al., 2021). However, while the associations between parental incarceration and children's mental and physical health are well established, the research is concentrated on the United States, where mass incarceration has greatly contributed to societal problems and health inequities (Bowleg, 2020; Lurigio, 2024) and where incarceration rates are notably higher than those in other developed democratic nations (Wildeman & Andersen, 2015). Therefore, there is a need for studies conducted in countries where offending is less likely to result in imprisonment (see, e.g., Roettger et al., 2022).

Another gap in the literature is the limited understanding of how less severe forms of parental offending may impact children (Hutson et al., 2023; Whitten et al., 2019), even though exposure to parental offending behavior is much more common than exposure to parental incarceration (e.g., Järvinen et al., 2024). A limited number of studies have examined parental offending behavior more broadly than just incarceration. However, such studies often encompass any parental criminal record or any involvement with the criminal justice system (Whitten et al., 2019), which makes it challenging to distinguish between the effects of minor versus serious offences. An exception to this is a study using Australian data that differentiated between any parental offending and violent offending and investigated their associations with early childhood physical health and well-being. The findings revealed that negative effects for children were most pronounced for those whose both parents were involved in offending and/or had committed violent offences, even when controlling for other socioeconomic risk factors (Laurens et al., 2017). Similarly, a study from Sweden that examined a broad range of child health and behavioral variables found that parental violent criminal convictions and imprisonment had stronger associations with children's outcomes compared to non-violent convictions and having no imprisonment (Järvinen et al., 2024).

The current study addresses the gaps in literature in several ways. Firstly, we examine data from Sweden, a high-income developed welfare democracy with lower income inequality and social segregation than the US where most previous research is concentrated. Further, the judicial system of Sweden differs significantly from that of the US, with incarceration rates in the past decades typically being one tenth of that in the US and sentence lengths considerably shorter, the median being around two months (see Grönqvist et al., 2024). Secondly, we conceptualize parental offending behavior as criminal convictions and distinguish between convictions from non-violent and violent offences. In 2024, 13% of all convictions in Sweden led to imprisonment, and these were dominated by drug offences, theft, and crimes against life and health (Brå - The Swedish National Council for Crime Prevention, 2025). Thus, our measure conflates parental incarceration (potentially present in both conviction categories) but is more nuanced than most previous research in that it distinguishes between parental violent and non-violent criminal behavior. Parental incarceration is associated with many repercussions that associate with parental absence, e.g., household instability, economic hardship and single parenting (Turney & Goodsell, 2018), making it difficult to disentangle their effects (see Wildeman et al., 2018 for a review assessing the causality of parental incarceration). Non-violent and violent offenders, in turn, seem to differ somewhat in personality and cognitive and executive functioning (Janes et al., 2024). These characteristics are heritable and may contribute to intergenerational transmission of hardships and are captured by our measure of parental offending. Thirdly, we examine both the mother's and the father's convictions to get a clearer picture on possible differences in the associations depending on parental gender. Fourthly, we examine child mortality, representing a severe correlate of parental criminal convictions, but is little studied because it requires large datasets for sufficient statistical power.

A limited number of prior studies have explored child mortality in relation to parental incarceration or other offending behaviors. These studies have found elevated all-cause mortality in early infancy in the U.S. (Wildeman, 2012), by the 18th birthday in Denmark (Ostergaard et al., 2019) and Sweden (Järvinen et al., 2024), and in adulthood in the Netherlands (van de Weijer et al., 2018), as well as higher external mortality by age five in children of parents with a police record in the Netherlands (Gaalen, 2016). Furthermore, a Danish study found higher mortality by age 20 in sons, but not daughters, of incarcerated parents (Wildeman et al., 2014). However, despite analyzing a full birth cohort of 59,000 children, their study was underpowered to detect all potential associations, since of the boys and girls who died during the follow-up period, only 30 and 5, respectively, had an incarcerated parent. This underscores the necessity of using very large samples to study outcomes as rare as child mortality in developed countries.

To the best of our knowledge, no studies have examined differences in the associations between parental offending behavior and child mortality across different childhood periods or explored specific causes of death in detail. Järvinen et al. (2024) distinguished between all deaths by age 18, and drug-related deaths, violent victimization, suicides, and other external causes, finding associations with all but drug-related deaths. However, their analyses did not account for other causes of death, such as disease deaths, for which the risk may also be elevated in children of offenders (see, e.g., findings on children's cardiovascular health or biomarkers of heightened stress levels: Boch & Ford, 2015; Del Toro et al., 2022; Roettger et al., 2022), and did not specify different developmental periods of children. The present study uses Swedish register data from all children born in Sweden between 1973 and 2013 (N = 4.2 million), including detailed information on causes of death and parental criminal convictions, categorized by type (violent vs. non-violent offending), to examine associations between parental convictions and child mortality in greater detail.

## 2 Methods

### 2.1 Data

The study employs several Swedish population-wide registers which were linked using the personal identity number assigned to all Swedes upon birth or immigration. The Total Population Register (Ludvigsson et al., 2016) and the Multi-Generation Register (Ekbom, 2011) include every individual born since 1932 and living in Sweden at any point since 1961, link them to their biological or adoptive parents, and were used to identify parents and offspring, their birth dates and parents’ country of birth. The sample includes all individuals born in Sweden in 1973–2013, for whom both parents could be identified (N = 4,198,952). Information on parental criminal convictions was obtained from the Swedish Crime Register (Brottsförebyggande rådet, 2024), which contains all convictions from the court of first instance from 1973 to 2013. Death dates and causes of death were available from 1 January 1973 and until 31 December 2013 in the Cause of Death Register (Brooke et al., 2017). Data on parental education and income were obtained from the National Censuses undertaken in 1970, 1975, 1980 and 1985, and from the Longitudinal Integration Database for Health Insurance and Labour Market Studies from 1990 onwards (Ludvigsson et al., 2019). Emigration dates were available in the Migration Register (Ludvigsson et al., 2016). Register linkages for the current study were approved by the Regional Ethical Review Board of Stockholm. The register-linkage was done by Swedish register authorities and pseudonymized before releasing it to the researchers. No informed consent was required for secondary use of pseudonymized register data. The data underlying this article were provided by different Swedish official registers under license for the current study. Data are available for all researchers after ethical vetting and application to the appropriate register holders.

### 2.2 Measures

Parental criminality included all convictions from the court of first instance and was categorized into ‘0’ No convictions (the reference category); ‘1’ Non-violent conviction(s) only; and ‘2’ at least one Violent conviction. Our division of non-violent and violent offences is based on whether direct aggressive behavior or threat against the life and health of another individual was involved. Violent convictions included (numbers within parentheses are law chapters and paragraphs of the Swedish Criminal Code, *brottsbalken, SFS 1962:700*) Homicide (3:1-3); (gross) Assault (3:5-6); Illegal threats (4:5; 4:7; 17:1-2); Coercion and offences against liberty and integrity (4:1-2, 4, 4a, 5, 7); (aggravated) Robbery (8:5-6); (aggravated) Arson (13:1-2); and Sexual crimes (excluding prostitution and the buying of sexual services; 6:1-6, 6:8-10, 6:12, 16:10A). Non-violent convictions included: Theft (8:1-2, 8:4, 8:7-10); Vandalism and sabotage (12:1-4; 13:3-10 (5a-b)); Unlawful entering of a person's home, Trespassing (4:6); Noncoercive sex with a relative (6:7); Fraud (9:1-10); Embezzlement (10:1-8 (5a-e)); Dishonesty/crime towards a creditor (11:1-5); Forgery (14:1-10); Road Traffic Offences (*Act 1951:649, Act 1972:603, and Act 1998:1276*); Narcotic offences (*Act 1968:649*); and other criminal convictions not elsewhere detailed.

Parental criminal convictions were included in the analyses irrespective of their timing. This was done because in the current study, a parental criminal conviction is seen as an indicator of risky parental behavior, and no direct causal association between parental criminality and child mortality is assumed. First registered parental convictions were concentrated around 10–15 years before the focal child's birth. 50 percent of maternal first convictions occurred between 9.0 years before and 5.7 years after the focal child's birth, and 50 percent of paternal first convictions between 12.4 years before and 1.3 years after the child's birth (see Supplementary Fig. S7–S8).

Cause of death was defined based on the underlying cause of death. We examined several causes of death: 1) All-cause mortality; 2) Deaths from diseases and medical conditions and their subcategories a) Communicable diseases and acute infections, b) Malign and benign neoplasms, c) Congenital disorders, and d) Other disease deaths; and 3) External-cause mortality and its subcategories a) Alcohol-related mortality, b) Accidents, c) Suicides, d) Homicides, and e) Other external causes (mainly external-cause mortality without clear indication of intentionality). The complete list of diagnoses included in each category is shown in Supplementary Table S1. Offspring and parent's sex was defined based on register information and coded as ‘0’ for females (the reference category) and ‘1’ for males. Parental education was divided into three categories, <9, 9 to <12 and 12+ years of education. Parental immigration status was coded as ‘0’ if the parent was born in Sweden (the reference category) and as ‘1’ if not. Missing information on parental education and immigration status were coded as additional categories and included in the analyses.

### 2.3 Statistical analyses

The associations between parental criminality and offspring mortality were examined by Cox proportional hazards models with attained age as the underlying timescale, separately for three periods: infancy (first year), childhood (ages 1–9), and adolescence (ages 10–19). Those not deceased contributed person-time at risk until their first, 10th, or 20th birthday (depending on the analysis period), the end of follow-up (end of 2013) or emigration, whichever occurred first. We ran separate models for the different causes of death (all-cause mortality and cause-specific models). In the cause-specific models, individuals were also censored if they died of another reason than the examined one (producing so-called cause-specific hazard ratios). The Efron method was used to handle tied failures. All models were adjusted for focal child sex and birth year and maternal age at the child's birth (and this in the second power to account for non-linear associations). To study the effects of parental criminality independent of some other related childhood risk factors, a second set of models further included the co-parent's criminality, parental education, and immigration status as covariates. The proportionality assumption of the Cox model was assessed by Schoenfeld residuals and visual examination of Kaplan-Meier curves (see Supplementary Text1 and Fig. S3–S6 for details). All analyses were carried out in Stata14.

### 2.4 Sensitivity analyses

Even though we treat parental criminal convictions as a risk marker, and not a causal prerequisite of child mortality, we conducted sensitivity analyses with a time-varying parental criminal conviction indicator to examine the role of timing of parental criminal convictions in the associations. This time-varying variable received value ‘0’ before the first conviction, ‘1’ after the first conviction if the parent did not have any violent crime convictions in the registers, and ‘2’ after the first conviction if the parent had at least one violent conviction in the registers (even if the first conviction was a non-violent one). We then repeated the main analyses for all-cause mortality in the different age periods with these time-varying variables as predictors.

Most child homicides are intrafamilial (Somander & Rammer, 1991). In case of homicide mortality, a parental violent criminal conviction might thus be directly related to the child's death (and not a marker of risky parental behavior that potentially increases child mortality risk), but these events could not be linked with certainty with the available register information. Of the 251 children who died of homicide in our sample, 59% did not, however, have a parent with a violent crime conviction. Nevertheless, to assess to what extent the possible overlap between parent's violent crime conviction and child's homicidal death affected our results, we conducted sensitivity analyses for all-cause mortality excluding children who died of homicide.

## 3 Results

The descriptive statistics of the sample by combined parental criminality are shown in Table 1. Altogether, 10.5% of the children had a mother with at least one non-violent crime conviction but no violent crime convictions and 1.2% had a mother with at least one conviction related to violent crime. The respective percentages for fathers were 30.1 % and 8.4 %. Compared to parents without criminal convictions, parents with convictions were more often born outside Sweden and were less educated. The mothers were also somewhat younger and had had their children at a younger age.

**Table 1:** Sample characteristics by parental criminal convictions.

	No parental convictions	Non-violent convictions	Violent convictions
N (%)	2,381,410	1,431,471	386,071
Deaths in infancy (n, %)	9500 (39.89)	6157 (43.01)	1802 (46.68)
Disease deaths	9241 (38.80)	5931 (41.43)	1717 (44.47)
External causes	91 (0.38)	102 (0.71)	51 (1.32)
Deaths in ages 1–9 (n, %)	2759 (11.58)	2026 (14.15)	681 (17.64)
Disease deaths	2071 (8.70)	1387 (9.69)	373 (9.66)
External causes	607 (2.55)	582 (4.07)	287 (7.43)
Deaths in ages 10–19 (n, %)	2558 (10.74)	1998 (13.96)	783 (20.28)
Disease deaths	1193 (5.01)	774 (5.41)	249 (6.45)
External causes	1307 (5.49)	1179 (8.24)	520 (13.47)
Mother's age at birth (mean, SD)	29.60 (5.00)	28.84 (5.37)	27.43 (5.66)
Mother's birth year (mean, SD)	1964 (12.16)	1964 (10.92)	1967 (11.00)
Father's birth year (mean, SD)	1961 (12.56)	1961 (11.23)	1963 (11.41)
Mother immigrant (%)	14.36	17.38	22.12
Father immigrant (%)	14.55	17.78	26.18
Mother's education (%)			
Low	9.63	14.05	22.36
Middle	41.88	49.49	54.11
High	47.44	35.69	22.39
Missing	1.06	0.77	1.14
Father's education (%)			
Low	14.47	20.71	30.49
Middle	44.34	52.44	55.64
High	40.05	25.96	12.60
Missing	1.15	0.89	1.27

Note. No parental criminality = Neither parent has any criminal convictions; Non-violent criminality = At least one of the parents has a non-violent crime conviction, but neither have violent crime convictions; Violent criminality = At least one of the parents has a violent crime conviction.

Disease deaths include communicable diseases and acute infections, malign and benign neoplasms, congenital disorders, and other disease deaths. External causes of death include alcohol-related mortality, accidents, suicides, homicides, and other external causes.

We first checked whether parental criminality was differently associated with offspring mortality for boys and girls by running models for all-cause mortality in all age periods with interaction terms between parental criminality and offspring sex. Boys had higher mortality in infancy (HR = 1.24, 95% CI = 1.21–1.28, p < .001), childhood (1.26, 1.20–1.33, p < .001), and adolescence (1.60, 1.52–1.69, p <. 001; reported for the fully adjusted models). The effects of parental criminality on offspring mortality were similar in boys and girls, except in ages 1–9, where maternal violent criminality was more strongly associated with sons' mortality than daughters' (interaction term's HR = 1.53, 95% CI 1.02–2.28, p = .037) and ages 10–19, where paternal violent criminality was also somewhat more strongly associated with sons' mortality (HR = 1.19, 1.00–1.41, p = .050). However, with the lack of systematic sex differences and to maintain statistical power with rare causes of death, all subsequent analyses were run for the whole sample, controlling for sex.

**Fig. 1:**
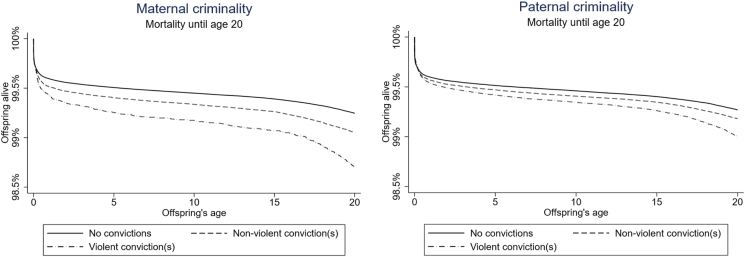
Observed survival curves (Kaplan-Meier curves) for all-cause mortality by maternal and paternal criminal convictions for the first 20 years of the children's lives.

By the first birthday, the cumulative incidence of death was 0.42%, by the 10th birthday, 0.55%, and by 20th birthday, 0.67%. Both maternal and paternal criminality were associated with a higher risk of death relative to children whose parents had no convictions (Fig. 1). The vast majority of all offspring deaths occurred within the first year of life, and mortality started increasing again slightly during puberty (Supplementary Fig. S1). In infancy and childhood, most offspring deaths were related to sudden infant death syndrome and diseases and medical conditions, such as congenital malformations and neoplasms. After the 10th birthday and especially among teenagers, external causes, such as accidents and suicides, became the most common causes of death (Supplementary Fig. S2).

**Fig. 2:**
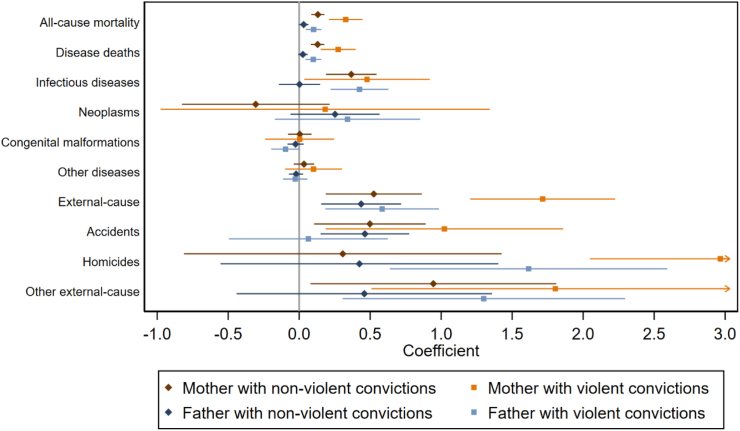
Child mortality in infancy by parental criminality. Coefficients from models adjusting for child's sex and birth year, maternal age at the child's birth, co-parent's criminality, and parental education and immigration status. All-cause mortality, disease deaths, and external-cause mortality include the respective sub-categories. “Other diseases” include other diseases and medical conditions. Hazard ratios are presented in Supplementary Table S2.

In infancy, by the first birthday, children's all-cause mortality was relatively higher for those with violent or non-violent maternal and paternal criminality (7–51% higher mortality risks), although the association with paternal non-violent criminality was modest in size (Supplementary Table S2). These associations were somewhat attenuated when controlling for co-parent's criminality, parental immigrant background and socioeconomic status, but remained statistically significant (Fig. 2 and Table S2; see results for all-cause mortality and covariates in Supplementary Table S5). The higher all-cause mortality in infancy among children of parents with criminal convictions consisted of higher mortality due to infections and communicable diseases, and higher accident, homicide, and other external-cause mortality. Homicide mortality was vastly increased among children of parents with violent crime convictions, and the estimates had extremely wide confidence intervals, indicating a small number of homicidal deaths in infancy coupled with a possibly high number of cases where the parent was the perpetrator of the homicide. This was investigated further in sensitivity analyses (see below and Supplementary Table S7). Mortality due to congenital malformations, neoplasms, or other diseases or medical conditions was not associated with parental criminality. In addition, paternal non-violent crime was not associated with any of the specific causes of death, especially when controlling for the additional risk factors, except for a higher risk of offspring accident mortality.

**Fig. 3:**
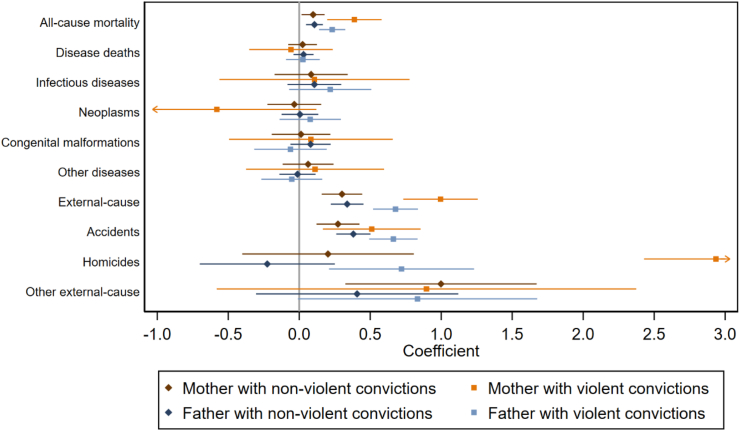
Child mortality in child ages 1–9, by parental criminality. Coefficients from models adjusting for child's sex and birth year, maternal age at the child's birth, co-parent's criminality, and parental education and immigration status. All-cause mortality, disease deaths, and external-cause mortality include the respective sub-categories. “Other diseases” include other diseases and medical conditions. Hazard ratios are presented in Supplementary Table S3.

In childhood, ages 1–9, parental non-violent and violent criminality were associated with relatively higher offspring all-cause mortality (17–76% higher mortality risk; Supplementary Table S3), also when controlling for parental education and immigrant status and co-parent's convictions (Fig. 3; Supplementary Table S3 and S5). Of the specific causes of death, all types of parental criminality were associated with higher offspring accident mortality, and parental violent criminality with higher offspring homicide mortality. Offspring of parents with criminal convictions also had higher other external mortality, but these associations were attenuated to a non-significant level in the fully adjusted models. Again, especially maternal violent criminality was very strongly associated with offspring homicidal mortality which was further examined in subsequent sensitivity analyses. Mortality due to any of the diseases or medical conditions was not associated with parental criminality, especially after controlling for the additional risk factors.

**Fig. 4:**
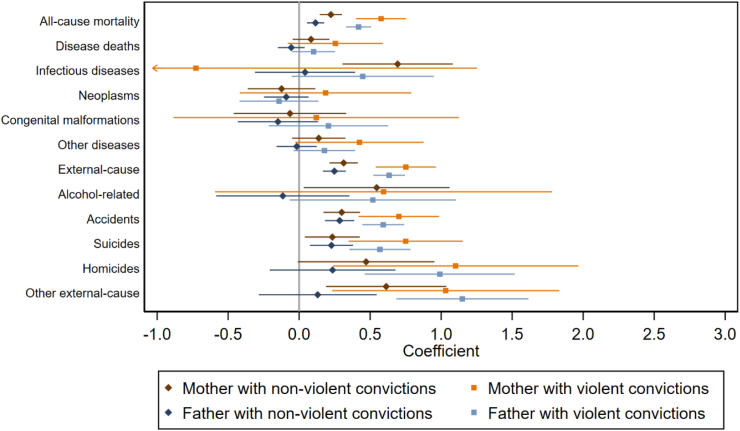
Child mortality in child ages 10–19, by parental criminality. Coefficients from models adjusting for child's sex and birth year, maternal age at the child's birth, co-parent's criminality, and parental education and immigration status. All-cause mortality, disease deaths, and external-cause mortality include the respective sub-categories. “Other diseases” include other diseases and medical conditions. Hazard ratios are presented in Supplementary Table S4.

In adolescence, ages 10–19, all types of parental criminality were associated with higher all-cause mortality in the offspring (18–117% higher mortality risk), even when controlling for the additional risk factors (Fig. 4; Supplementary Table S4 and S5). Specifically, all types of parental criminality were associated with higher accident and suicide mortality, all but paternal non-violent criminality were associated with higher other external mortality, maternal and paternal violent criminality were associated with higher homicide mortality, and maternal non-violent criminality was associated with higher mortality related to infectious diseases and alcohol. Mortality due to neoplasms, congenital malformations, or other diseases and medical conditions was not associated with parental criminal convictions.

### 3.1 Statistical checks and sensitivity analyses

The proportionality assumption of Cox regression was assessed by Schoenfeld residuals and by visual comparison of the Kaplan-Meier survival curves (supplementary Text 1 and Fig. S3–S6). Based on these examinations, we concluded that the proportionality assumption was met in the scope of the current study.

Our measure for parental criminality encompasses all convictions, irrespective of their timing. To see if this broad definition impacted the results, we conducted sensitivity analyses where parental criminality was defined time-varyingly, being ‘0’ until the first conviction. The results of these analyses were largely comparable to the main analyses, with one estimate losing statistical significance (maternal violent convictions and childhood mortality) and one gaining it (paternal non-violent convictions and mortality in infancy) in the models adjusting for additional risk factors (Supplementary Table S6). The timing of parental criminal convictions thus had little effect on the associations.

Parental, especially maternal, violent criminality was associated with vastly increased relative risk of homicide mortality in the offspring (Fig. 2, Fig. 3, Fig. 4; Supplementary Table S2–S4), but as stated above, this association can be driven by the parent being convicted of the said filicide. To examine to what extent these strong associations affected the overall association between parental criminality and offspring all-cause mortality, we ran models for all-cause mortality excluding cases where homicide was the underlying cause of death. In these models, the associations between parental criminality and child all-cause mortality were practically identical to the main models in infancy (Supplementary Table S7). In childhood, all other associations were little changed, but the association between maternal violent criminality and offspring mortality was markedly attenuated, and in the model controlling for additional risk factors, rendered statistically non-significant (Table S7). Thus, in childhood, parental non-violent convictions and paternal violent convictions were robustly associated with offspring all-cause mortality, while maternal violent convictions were only robustly associated with accident mortality. In ages 10–19, associations between parental criminality and child all-cause mortality were, again, only marginally affected by excluding homicidal deaths (Table S7).

## 4 Discussion

In line with previous research showing parental offending behavior being associated with compromised child health (Hutson et al., 2023; Whitten et al., 2019; Wildeman et al., 2018) and mortality (Järvinen et al., 2024; Ostergaard et al., 2019; van de Weijer et al., 2018; Wildeman, 2012; Wildeman et al., 2014), we found parental violent and non-violent criminal convictions to be associated with children's higher mortality throughout childhood and adolescence. Our study elaborates previous findings by examining the risks in different age periods, investigating detailed causes of death and separating between parental non-violent and violent convictions, and by looking at both the mother's and father's criminal records.

The associations varied somewhat between different ages. In infancy, parental convictions were associated with children's higher mortality due to infections and communicable diseases, and higher accident and other external mortality, whereas in ages 1–9, they were mainly associated with accident mortality, but not other external nor disease deaths. In ages 10–19, mortality due to infections was again elevated among children with a mother who had non-violent conviction(s), and all types of parental convictions were associated with higher accident and other external mortality and with a higher likelihood of committing suicide. The associations were generally larger with respect to mother's convictions compared to the father's. In addition, our results clearly show a “dose-response” pattern in child mortality: mortality among children whose parents had no criminal convictions was lower than among children whose parents had non-violent convictions, which in turn was lower than mortality among children whose parents were convicted of more serious, violent crimes.

Our study is largely descriptive in that it only shows associations between parental convictions and child mortality, treats parental convictions as a marker for the tendency of the parent to behave in a risky way, and assumes no direct causal effect of parental criminal convictions on child mortality. Causal links may exist, but parental criminal convictions can also be associated with a risky rearing environment that could increase child mortality even in the absence of parental criminal behavior (see Jackson et al., 2021 for a study on parental incarceration and related childhood adversities). Controlling for some of these additional risk factors (low parental education, parental immigrant status, and the co-parent's criminal convictions) attenuated some of the associations found in our study, but they still mostly remained statistically significant. Our sensitivity analyses accounting for the timing of parental criminal convictions also points to the possibility of non-causal associations between parental criminal behavior and child mortality. With the results of these sensitivity analyses being virtually identical with the main analyses (where parent's first conviction could occur even after the child's death), it seems that children's mortality risk could be elevated irrespective of the timing of the parent's offending behavior.

Existing research provides possible pathways between parental criminal convictions and higher offspring mortality due to infectious diseases in infancy found in this study. Both the mother's and the father's criminal behavior and incarceration have been shown to be associated with the mother's sub-optimal health behavior and lack of medical screening during pregnancy, which in turn are associated with poorer child and mother perinatal health (Dumont et al., 2014; Lee et al., 2023; Testa et al., 2020; Yi et al., 2021). Further, parental criminality may be associated with disorganization and lack of routine in family life, which compromises parents' ability to attend to their children's medical needs (Jenny et al., 2007). Such circumstances can, in extreme situations, result in the heightened infectious disease mortality found among infants and adolescents in this study. Another possible pathway between parental criminality and offspring infectious mortality is that children of offenders exhibit signs of chronic stress (Boch & Ford, 2015; Del Toro et al., 2022), which compromises the functioning of the immune system (Goin, 2011). 99% of children aged 0–6 in Sweden attend regular health care visits in the Child Health Services (bhvq.se/in-english/). Further research on the mechanisms of the found associations is needed, but our results point to the need for developing these services. Future improvements could include, for example, early parental risk screening and enhanced vaccination counseling and intensified collaboration with Social Services. Since our results show an elevated infectious disease mortality also in adolescence in children of convicted parents, intensified multidisciplinary support could strengthen the continuity of care and address behavioral or environmental risk factors that compromise child health in the long run.

In this study, children of parents with criminal convictions were also more likely to die of external causes throughout childhood and adolescence. These observed associations are likely to manifest several different mechanisms. In infancy and early childhood, higher accident and other external mortality can result from the parents' behavior. Offending behavior is highly comorbid with traits such as attention deficit/hyperactivity disorder and substance use disorders (Knecht et al., 2015) that increase the likelihood of accidents (Nigg, 2013), compromise parenting (Park et al., 2017), and are associated with higher offspring mortality (Berg et al., 2022). As children age and gain more independence, their own behavior becomes an increasingly important factor in accident mortality. Since offending behavior, and its correlates such as ADHD, substance abuse, and impulsivity are largely genetically driven (Pezzoli et al., 2025), parents with criminal convictions are more likely than other parents to pass on such tendencies to their children, which in turn increases the children's likelihood of accident injuries and mortality (Dalsgaard et al., 2015). However, since child mortality is – fortunately – a very rare event in contemporary Sweden, we could not conduct analyses that would consider the shared genetic background of parents and children (McAdams et al., 2014). Future studies should utilize even larger datasets and genetically informative study designs to further investigate the intergenerational mechanisms between parental criminality and child mortality.

Finally, an important and alarming finding was that the risk of suicide mortality in ages 10–19 was 26–112% higher in children of parents with criminal convictions compared to other children. Previous research has pointed out that the mental health of children of offenders is worse (Whitten et al., 2019), and according to our study, this seems to sometimes have extreme consequences. Research on improving mental health or preventing suicides of children of parents with criminal convictions is currently limited, but interventions that focus on positive parenting techniques, attachment, and family functioning show some promise in potentially aiding this vulnerable group (Berkel et al., 2023; Machell et al., 2016; Merhi et al., 2024). However, the positive effects tend to wear off, if interventions are short-term (Merhi et al., 2024), stressing the need for continuity of care and support, collaboration between social and health services, as well as comprehensive and long-term interventions.

By utilizing a very large register sample of 4.2 million children we were able to examine mortality and causes of deaths in children of parents with criminal convictions in greater detail than in previous literature. We found that the associations between parental convictions and child mortality persisted even when controlling for some other childhood risk factors such as low parental education. However, even with this dataset, some causes of death were very few in number, restricting the possibility to add a more comprehensive list of possible confounders or to further detail the main predictors (e.g., comparing repeated vs. one-time-only parental convictions). Parental offending behavior or incarceration often coincide with other adverse childhood experiences (Björkenstam et al., 2017; Lanier et al., 2018), which is why their unique role in child mortality deserves more attention. Earlier literature is somewhat inconsistent in whether parental criminality is an independent risk factor for child well-being above and beyond other associated childhood adversities (see e.g., Jackson et al., 2021; van de Weijer et al., 2018). Future studies with even larger datasets, for example, combining data from other Nordic countries with similarly extensive registers, could allow for a more nuanced investigation of parental criminal convictions or take into account a more exhaustive list of childhood adversities. Another possibility for future research on somewhat smaller datasets would be focusing on all-cause mortality and more detailed predictors and confounders.

A further caveat of this study is that while using register information on parental convictions allowed for differentiating between violent and non-violent offending, we only had information on behavior that led to involvement with the criminal justice system. In the case of more serious crimes this is likely to be a smaller problem, but minor offences more easily go undetected by the judicial system. Thus, it is likely that our “control group”, parents with no criminal convictions, includes a fair number of individuals who have behaved delinquently at some point in their lives without being caught. This makes our estimates conservative, since the “control group” also includes children who have some of the same parental risk factors as the “treatment group”.

One third of our sample had at least one parent with a non-violent criminal conviction, and almost one in ten children a parent with a violent criminal conviction. They are not a small group, and our findings indicate a heightened risk for the most serious of outcomes for these children throughout their childhood and adolescence. We show elevated risks for multiple causes of death, ranging from disease deaths to accidents and suicides. Sweden is a highly developed Nordic society with a strong welfare system, but our results imply that children in such high-risk families still need more attention and long-term services that range from support for parenting to programs aimed at diminishing risky behavior and improving mental health for the children of offenders.

## CRediT authorship contribution statement

**Venla Berg:** Writing – review & editing, Writing – original draft, Methodology, Formal analysis, Conceptualization. **Ralf Kuja-Halkola:** Writing – review & editing, Methodology, Data curation, Conceptualization. **Henrik Larsson:** Writing – review & editing, Methodology, Data curation, Conceptualization. **Paul Lichtenstein:** Writing – review & editing, Data curation, Conceptualization. **Antti Latvala:** Writing – review & editing, Supervision, Funding acquisition, Conceptualization.

## Ethical statement

The authors state that they have followed the ethical guidelines stated in Elsevier's Publishing Ethics Policy.

Register linkages for the current study were approved by the Regional Ethical Review Board of Stockholm. No informed consent was required for secondary use of anonymized register data. The data underlying this article were provided by different Swedish official registers under license for the current study. Data are available for all researchers after ethical vetting and application to the appropriate register holders.

## Funding

This study is funded by the Research Council of Finland (Decision Numbers: 308698, 335589 and 339646 to AL) and Strategic Research Council established within the Research Council of Finland (Decision Numbers: 364382 and 364371 to VB).

## Declaration of interest

HL reports receiving grants and personal fees from Shire/Takeda and personal fees from Evolan, all outside the submitted work. The other authors declare no competing interests.

## Data Availability

The authors do not have permission to share data.

## References

[bib1] Berg V., Kuja-Halkola R., Khemiri L., Larsson H., Lichtenstein P., Latvala A. (2022). Parental alcohol and drug abuse and offspring mortality by age 10: A population-based register study. The European Journal of Public Health.

[bib2] Berkel C., Hara K.O., Eddy J.M., Rhodes C.A., Blake A., Thomas N., Hita L., Herrera D., Wheeler A.C., Wolchik S. (2023). The prospective effects of caregiver parenting on behavioral health outcomes for children with incarcerated parents : A family resilience perspective. Prevention Science.

[bib3] Björkenstam C., Kosidou K., Björkenstam E. (2017). Childhood adversity and risk of suicide: Cohort study of 548 721 adolescents and young adults in Sweden. BMJ.

[bib4] Boch S.J., Ford J.L. (2015). C-Reactive protein levels among U.S. adults exposed to parental incarceration. Biological Research For Nursing.

[bib5] Bomysoad R.N., Francis L.A. (2022). Associations between parental incarceration and youth mental health conditions: The mitigating effects of adolescent resilience and positive coping strategies. Current Psychology.

[bib6] Bowleg L. (2020). Reframing mass incarceration as a social-structural driver of health inequity. American Journal of Public Health.

[bib7] Brå - The Swedish National Council for Crime Prevention (2025). Statistics from the judicial system. https://Bra.Se/English/Statistics/Statistics-from-the-Judicial-System.

[bib8] Brooke H.L., Talbäck M., Hörnblad J., Johansson L.A., Ludvigsson J.F., Druid H., Feychting M., Ljung R. (2017). The Swedish cause of death register. European Journal of Epidemiology.

[bib9] Dalsgaard S., Ostergaard S.D., Leckman J.F., Mortensen P.B., Pedersen M.G. (2015). Mortality in children, adolescents, and adults with attention deficit hyperactivity disorder: A nationwide cohort study. The Lancet.

[bib10] Del Toro J., Fine A., Wang M.T., Thomas A., Schneper L.M., Mitchell C., Mincy R.B., McLanahan S., Notterman D.A. (2022). The longitudinal associations between paternal incarceration and family well-being: Implications for ethnic/racial disparities in health. Journal of the American Academy of Child & Adolescent Psychiatry.

[bib11] Dumont D.M., Wildeman C., Lee H., Gjelsvik A., Valera P., Clarke J.G. (2014). Incarceration, maternal hardship, and perinatal health behaviors. Maternal and Child Health Journal.

[bib12] Ekbom A., Dillner J. (2011).

[bib13] Gaalen R. van (2016). Parental crime and the safety and survival of small children. Advances in Life Course Research.

[bib14] Goin J.-P. (2011). Chronic stress, immune dysregulation, and health. American Journal of Lifestyle Medicine.

[bib15] Grönqvist H., Niknami S., Palme M., Priks M. (2024). The intergenerational effects of parental incarceration. IFN Working Paper).

[bib16] Haskins A.R. (2015). Paternal incarceration and child-reported behavioral functioning at age 9. Social Science Research.

[bib17] Herreros-Fraile A., Carcedo R.J., Viedma A., Ramos-Barbero V., Fernández-Rouco N., Gomiz-Pascual P., del Val C. (2023). Parental incarceration, development, and well-being: A developmental systematic review. International Journal of Environmental Research and Public Health.

[bib18] Hutson T., Thurman W., Kim M., Heitkemper E. (2023). Investigating the health and health-related quality of life impacts of the criminal legal system on families: A scoping review. Journal of Health Care for the Poor and Underserved.

[bib19] Jackson D.B., Testa A., Semenza D.C., Vaughn M.G. (2021). Parental incarceration, child adversity, and child health: A strategic comparison approach. International Journal of Environmental Research and Public Health.

[bib20] Janes S., McIntosh L.G., O'Rourke S., Schwannauer M. (2024). Examining the cognitive contributors to violence risk in forensic samples: A systematic review and meta-analysis. Aggression and Violent Behavior.

[bib21] Järvinen A., Lichtenstein P., D'Onofrio B.M., Fazel S., Kuja-Halkola R., Latvala A. (2024). Health, behavior, and social outcomes among offspring of parents with criminal convictions: A register-based study from Sweden. Journal of Child Psychology and Psychiatry.

[bib22] Jenny C., Christian C., Hibbard R.A., Kellogg N.D., Spivak B.S., Stirling J., Corwin D.L., Mercy J., Hurley T.P. (2007). Recognizing and responding to medical neglect. Pediatrics.

[bib23] Jolliffe D., Farrington D.P., Piquero A.R., MacLeod J.F., van de Weijer S. (2017). Prevalence of life-course-persistent, adolescence-limited, and late-onset offenders: A systematic review of prospective longitudinal studies. Aggression and Violent Behavior.

[bib24] Knecht C., De Alvaro R., Martinez-Raga J., Balanza-Martinez V. (2015). Attention-deficit hyperactivity disorder (ADHD), substance use disorders, and criminality: A difficult problem with complex solutions. International Journal of Adolescent Medicine and Health.

[bib25] Lanier P., Maguire-Jack K., Lombardi B., Frey J., Rose R.A. (2018). Adverse childhood experiences and child health outcomes: Comparing cumulative risk and latent class approaches. Maternal and Child Health Journal.

[bib26] Laurens K.R., Tzoumakis S., Kariuki M., Green M.J., Hamde M., Harris F., Carr V.J., Dean K. (2017). Pervasive influence of maternal and paternal criminal offending on early childhood development: A population data linkage study. Psychological Medicine.

[bib27] Lee R.D., D'Angelo D.V., Dieke A., Burley K. (2023). Recent incarceration exposure among parents of live-born infants and maternal and child health. Public Health Reports.

[bib28] Ludvigsson J.F., Almqvist C., Bonamy A.E., Neovius M., Ljung R., Michae K., Stephansson O., Ye W. (2016). Registers of the Swedish total population and their use in medical research. European Journal of Epidemiology.

[bib29] Ludvigsson J.F., Svedberg P., Olén O., Bruze G., Neovius M. (2019). The longitudinal integrated database for health insurance and labour market studies (LISA) and its use in medical research. European Journal of Epidemiology.

[bib30] Lurigio A.J. (2024). The golden anniversary of mass incarceration in America.

[bib31] Machell K.A., Rallis B.A., Esposito-Smythers C. (2016). Family environment as a moderator of the association between anxiety and suicidal ideation. Journal of Anxiety Disorders.

[bib32] McAdams T.A., Neiderhiser J.M., Rijsdijk F.V., Narusyte J., Lichtenstein P., Eley T.C. (2014). Accounting for genetic and environmental confounds in associations between parent and child characteristics: A systematic review of children-of-twins studies. Psychological Bulletin.

[bib33] Merhi D., Demou E., Niedzwiedz C. (2024). Mental health and behavioral interventions for children and adolescents with incarcerated parents: A systematic review. Journal of Child and Family Studies.

[bib34] Nigg J.T. (2013). Attention-deficit/hyperactivity disorder and adverse health outcomes. Clinical Psychology Review.

[bib35] Ostergaard S.D., Larsen J.T., Petersen L., Smith G.D., Agerbo E. (2019). Psychosocial adversity in infancy and mortality rates in childhood and adolescence: A birth cohort study of 1.5 million individuals. Epidemiology.

[bib36] Park J.L., Hudec K.L., Johnston C. (2017). Parental ADHD symptoms and parenting behaviors: A meta-analytic review. Clinical Psychology Review.

[bib37] Pezzoli P., McCrory E.J., Viding E. (2025). Shedding light on antisocial behavior through genetically informed research. Annual Review of Psychology.

[bib38] rådet B. (2024). Swedish crime statistics. https://bra.se/bra-in-english/home.html.

[bib39] Roettger M.E., Houle B., Najman J., McGee T.R. (2022). Parental imprisonment as a risk factor for cardiovascular and metabolic disease in adolescent and adult offspring: A prospective Australian birth cohort study. SSM, Population Health.

[bib40] Somander L.K.H., Rammer L.M. (1991). Intra- and extrafamilial child homicide in Sweden 1971-1980. Child Abuse & Neglect.

[bib41] Testa A., Jackson D.B., Vaughn M.G., Bello J.K. (2020). Incarceration as a unique social stressor during pregnancy: Implications for maternal and newborn health. Social Science & Medicine.

[bib42] Tolliver D.G., Hawks L.C., Holaday L.W., Wang E.A. (2024). Exploring parental incarceration, US government support programs, and child health and well-being: A national cross-sectional study. Journal of Pediatrics.

[bib43] Turney K. (2022). Chains of adversity: The time-varying consequences of paternal incarceration for adolescent behavior. Journal of Quantitative Criminology.

[bib44] Turney K., Goodsell R. (2018). Parental incarceration and children's wellbeing. The Future of Children.

[bib45] van de Weijer S.G.A., Smallbone H.S., Bouwman V. (2018). Parental imprisonment and premature mortality in adulthood. Journal of Developmental and Life-Course Criminology.

[bib46] Whitten T., Burton M., Tzoumakis S., Dean K. (2019). Parental offending and child physical health, mental health, and drug use outcomes: A systematic literature review. Journal of Child and Family Studies.

[bib47] Wildeman C. (2012). Imprisonment and infant mortality. Social Problems.

[bib48] Wildeman C., Andersen L.H. (2015). Cumulative risks of paternal and maternal incarceration in Denmark and the United States. Demographic Research.

[bib49] Wildeman C., Andersen S.H., Lee H., Karlson K.B. (2014). Parental incarceration and child mortality in Denmark. American Journal of Public Health.

[bib50] Wildeman C., Goldman A.W., Turney K. (2018). Parental incarceration and child health in the United States. Epidemiologic Reviews.

[bib51] Yi Y., Kennedy J., Chazotte C., Huynh M., Jiang Y., Wildeman C. (2021). Paternal jail incarceration and birth outcomes: Evidence from New York City, 2010–2016. Maternal and Child Health Journal.

[bib52] Zoutewelle-Terovan M., Skardhamar T. (2016). Timing of change in criminal offending around entrance into parenthood: Gender and cross-country comparisons for At-Risk individuals. Journal of Quantitative Criminology.

