# Supplementary material for: Mortality in offspring with parental criminal convictions: A population-based register study from Sweden
Source: SSM Popul Health. 2026 May 2;34:101928. doi: 10.1016/j.ssmph.2026.101928 (PMC13195284; doi:10.1016/j.ssmph.2026.101928)
Supplement: Multimedia component 1 [file mmc1.pdf]

Supplementary material for

Parental criminal convictions are associated with offspring mortality: A population-based register study from Sweden

Berg, Venla*[a,b]; Kuja-Halkola, Ralf[b]; Larsson, Henrik[b, d]; Lichtenstein, Paul[b]; Latvala, Antti[b,c]

[a]Population Research Institute, Väestöliitto (the Family Federation of Finland), Finland

[b]Department of Medical Epidemiology and Biostatistics, Karolinska Institutet, Sweden

[c]Institute of Criminology and Legal Policy, University of Helsinki, Finland

[d]School of Medical Sciences, Örebro University, Sweden

*Corresponding author

Venla Berg
email: venla.berg@vaestoliitto.fi
tel. +358 50 369 6429
Address: Väestöliitto, P.O.Box 849, FI 00100 Helsinki, Finland

Funding: This study is funded by the Research Council of Finland (Decision Numbers: 308698, 335589 and 339646 to AL) and Strategic Research Council established within the Research Council of Finland (Decision Numbers: 364382 and 364371 to VB).

Declaration of interest: HL reports receiving grants and personal fees from Shire/Takeda and personal fees from Evolan, all outside the submitted work. The other authors declare no competing interests.

Table S1. ICD codes used to classify the causes of death.

	ICD 8	ICD 9	ICD 10
**Causes of death**			
**Disease deaths**			
Sudden infant death syndrome	795,00; 796,20	798A	R95
Infections and communicable diseases	000-134*; 136*; 275,9*; 320*; 323*; 46-48*; 528,1*	001-040A; 040C-099B; 099D-134*; 136B-139*; 320-326*; 460-466*; 480-487*; 771C; 790H	A*; B*; J00-J06*; J09-J18*; J20-J22*; J65*; J84.9; G00-G09*
Neoplasms, malign and benign	140-239*; 275,50	140-237G; 237X-239*; 273C	C00-D48*
Congenital malformations	74-759*	237H; 740-759*	Q00-Q85*; Q86.1-Q99*
Other diseases	135*; 240-290*; 292-302*; 304-315*; 321-322*; 324-458*; 490-528,0; 528,2-570*; 571,9-738*; 760-794*	040B; 099C; 135-136A; 240-273B; 273D-290*; 292-302*; 304*; 306-319*; 330-357E; 357G-425E; 425H-459*; 467-478*; 490-535C; 535E-570*; 571E-739; 760A-H; 760X-779E; 779G-790C; 790E-797*; 798B-X; 799A	D50-D89*; E*; F00-F09*; F11-F99*; G10-G31.1; G31.8-G62.0; G62.2-G72.0; G72.2-I42.5; I42.7-I99*; J23-J64*; J66-J84.8; J85-K29.1; K29.3-K67*; K71-K85.1; K85.3-K85.9; K86.1-O35.3; O35.5-P04.2; P04.4-P96*; R00-R53-R94*
**External-cause mortality**			
Alcohol-related causes	291*; 303*; 571,00; 571,01; E860	291*; 303*; 305A*; 305X; 357F; 425F; 353D; 571A-D; 760W; 779F; E860*	F10*; G31.2; G62.1; G72.1; I42.6; K29.2; K70*; K85.2*; K86.0; O35.4*; P04.3; Q86.0; X45; Y15
Accidents	E800-E859; E861–E946; E948	E800–E858; E861–E869; E880–E929	V01–X44*; X46–X59*; Y85–Y86*
Suicides	E95*	E95*	X60–X84; Y87.0
Homicides	E96*	E96*	X85-Y09*; Y87.1
Other external causes	E930-E936; E947; E949; E97*–E99*	E87*; E93–E94*; E97*– E99*	Y10–Y14*; Y16–Y84*; Y87.2; Y88–Y89*

Note. Classification of causes of death is based on the classification of Statistics Finland (available at https://stat.fi/fi/luokitukset/kuolinsyyt/kuolinsyyt_80_20210101/; accessed on 6 November 2023), with some categories combined and modified to Swedish ICD codes. *Any code starting with the said.

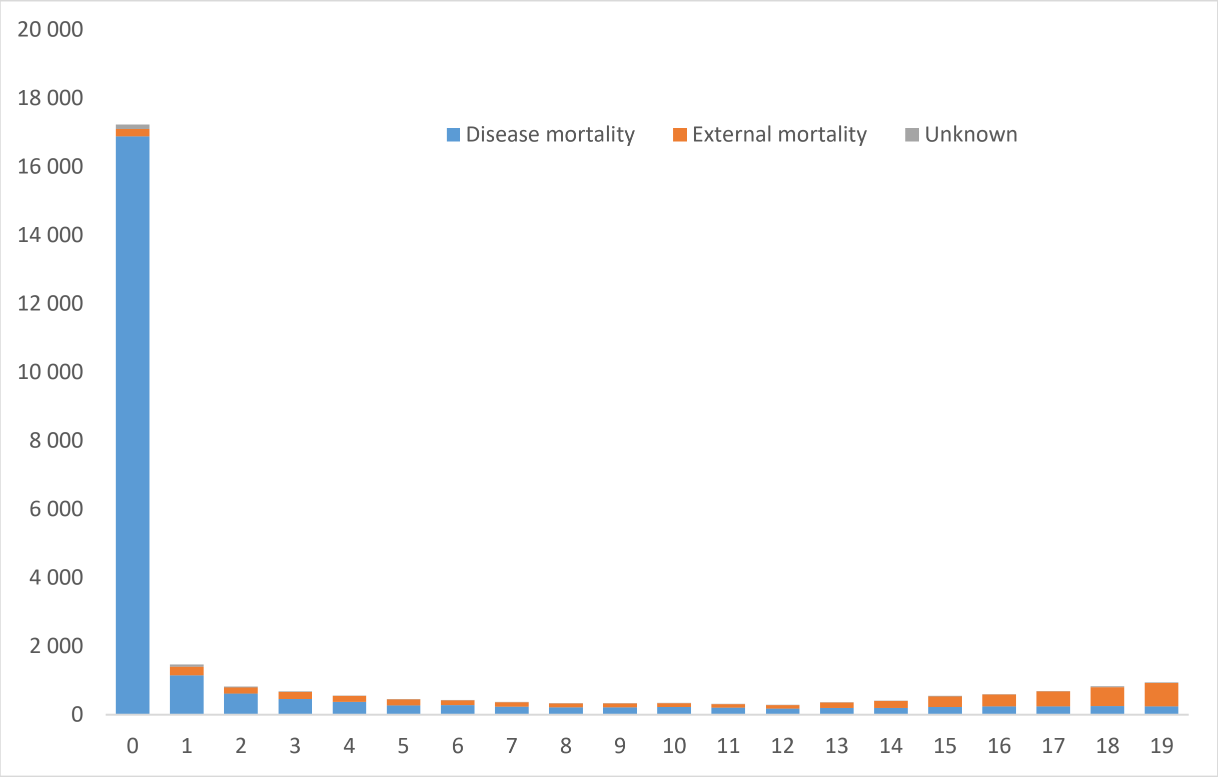

Figure S1. Number of deaths by age at death and underlying cause of death in the whole sample.

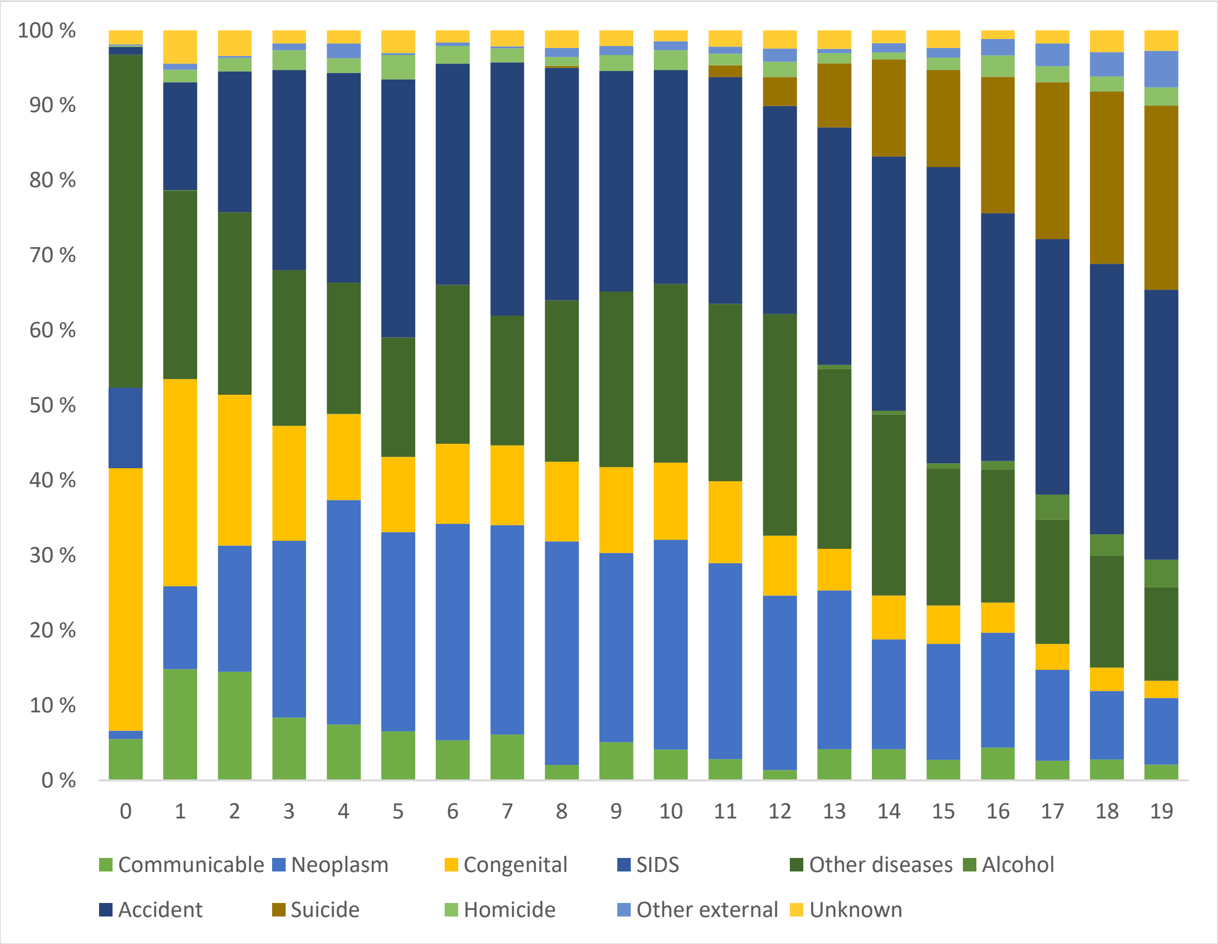

Figure S2. Proportions of different causes of death by age at death in the whole sample.

Tables S2–S4 show results (hazard ratios) from the main analyses, i.e., Cox regressions predicting all-cause and cause-specific mortality by parental ciriminal convictions in infancy, ages 1–9, and ages 10–19, respectively.

Table S2. Hazard ratios (HR) and 95% CIs from Cox regressions predicting offspring all-cause and cause-specific mortality in infancy by parental criminal convictions.

		Model 1		Model 2	
		HR (95%CI)	p	HR (95%CI)	p
**All-cause mortality**					
Maternal convictions	Non-violent	**1.19 (1.14, 1.25)**	**<.001**	**1.14 (1.09, 1.19)**	**<.001**
	Violent	**1.51 (1.35, 1.70)**	**<.001**	**1.39 (1.23, 1.56)**	**<.001**
Paternal convictions	Non-violent	**1.07 (1.04, 1.11)**	**<.001**	1.03 (1.00, 1.07)	.063
	Violent	**1.21 (1.15, 1.28)**	**<.001**	**1.11 (1.05, 1.17)**	**<.001**
**Disease deaths**					
Maternal convictions	Non-violent	**1.18 (1.13, 1.24)**	**<.001**	**1.14 (1.09, 1.19)**	**<.001**
	Violent	**1.43 (1.27, 1.62)**	**<.001**	**1.32 (1.16, 1.49)**	**<.001**
Paternal convictions	Non-violent	**1.06 (1.03, 1.10)**	**<.001**	1.03 (0.99, 1.06)	.129
	Violent	**1.20 (1.14, 1.27)**	**<.001**	**1.10 (1.04, 1.17)**	**<.001**
Infectious diseases					
Maternal convictions	Non-violent	**1.59 (1.33, 1.89)**	**<.001**	**1.44 (1.21, 1.72)**	**<.001**
	Violent	**2.01 (1.30, 3.10)**	**.002**	**1.61 (1.04, 2.51)**	**.034**
Paternal convictions	Non-violent	1.07 (0.93, 1.23)	.365	1.00 (0.87, 1.16)	.974
	Violent	**1.79 (1.48, 2.18)**	**<.001**	**1.53 (1.25, 1.87)**	**<.001**
Neoplasms					
Maternal convictions	Non-violent	0.79 (0.47, 1.32)	.375	0.74 (0.44, 1.24)	.249
	Violent	1.39 (0.44, 4.37)	.572	1.20 (0.38, 3.83)	.757
Paternal convictions	Non-violent	1.29 (0.95, 1.76)	.105	1.29 (0.94, 1.76)	.115
	Violent	1.41 (0.86, 2.32)	.171	1.41 (0.84, 2.35)	.192
Congenital malformations					
Maternal convictions	Non-violent	1.01 (0.93, 1.10)	.734	1.00 (0.92, 1.09)	.920
	Violent	1.01 (0.80, 1.29)	.921	1.00 (0.79, 1.28)	.976
Paternal convictions	Non-violent	1.00 (0.94, 1.06)	.976	0.97 (0.92, 1.03)	.379
	Violent	0.96 (0.87, 1.05)	.360	0.91 (0.82, 1.00)	.061
Other diseases					
Maternal convictions	Non-violent	1.06 (0.99, 1.14)	.097	1.03 (0.96, 1.11)	.360
	Violent	1.16 (0.95, 1.42)	.133	1.11 (0.90, 1.35)	.325
Paternal convictions	Non-violent	1.01 (0.96, 1.06)	.822	0.98 (0.93, 1.03)	.400
	Violent	1.04 (0.95, 1.13)	.393	0.97 (0.89, 1.06)	.525
**External-cause mortality**					
Maternal convictions	Non-violent	**1.94 (1.40, 2.70)**	**<.001**	**1.69 (1.20, 2.37)**	**.002**
	Violent	**7.04 (4.31, 11.50)**	**<.001**	**5.56 (3.34, 9.26)**	**<.001**
Paternal convictions	Non-violent	**1.66 (1.26, 2.19)**	**<.001**	**1.55 (1.17, 2.05)**	**.002**
	Violent	**2.36 (1.62, 3.44)**	**<.001**	**1.79 (1.20, 2.68)**	**.004**
Accidents					
Maternal convictions	Non-violent	**1.78 (1.22, 2.62)**	**.003**	**1.65 (1.11, 2.44)**	**.013**
	Violent	**3.06 (1.35, 6.97)**	**.008**	**2.78 (1.20, 6.42)**	**.017**
Paternal convictions	Non-violent	**1.66 (1.22, 2.25)**	**.001**	**1.59 (1.16, 2.17)**	**.004**
	Violent	1.26 (0.74, 2.16)	.400	1.07 (0.61, 1.87)	.820

Homicides					
Maternal convictions	Non-violent	1.89 (0.63, 5.62)	.255	1.36 (0.44, 4.16)	.591
	Violent	**35.83 (15.54, 82.64)**	**<.001**	**19.42 (7.75, 48.70)**	**<.001**
Paternal convictions	Non-violent	1.74 (0.67, 4.53)	.254	1.53 (0.57, 4.07)	.395
	Violent	**9.73 (4.06, 23.32)**	**<.001**	**5.03 (1.89, 13.37)**	**.001**
Other external-cause					
Maternal convictions	Non-violent	**3.37 (1.46, 7.80)**	**.005**	**2.57 (1.08, 6.11)**	**.032**
	Violent	**9.64 (2.77, 33.56)**	**<.001**	**6.08 (1.66, 22.19)**	**.006**
Paternal convictions	Non-violent	1.85 (0.77, 4.47)	.171	1.58 (0.64, 3.89)	.317
	Violent	**5.24 (2.07, 13.28)**	**<.001**	**3.67 (1.36, 9.92)**	**.010**

Note. Reference category for convictions: No convictions. There were no alcohol-related deaths or suicides in infancy.
Model 1 controls for offspring's sex, mother's age at the child's birth and this in the second power, and child’s birth year. Model 2 controls for Model 1 and the co-parent’s criminality, parental education, and parental immigration status.
Disease deaths and external-cause mortality include all the respective sub-categories.

Table S3. Hazard ratios (HR) and 95% CIs from Cox regressions predicting offspring all-cause and cause-specific mortality in ages 1–9 by parental criminal convictions.

		Model 1		Model 2	
		HR (95%CI)	p	HR (95%CI)	p
**All-cause mortality**					
Maternal convictions	Non-violent	**1.20 (1.11, 1.30)**	**<.001**	**1.10 (1.02, 1.20)**	**.019**
	Violent	**1.76 (1.46, 2.13)**	**<.001**	**1.47 (1.22, 1.79)**	**<.001**
Paternal convictions	Non-violent	**1.17 (1.10, 1.24)**	**<.001**	**1.11 (1.05, 1.18)**	**<.001**
	Violent	**1.43 (1.31, 1.56)**	**<.001**	**1.26 (1.15, 1.38)**	**<.001**
**Disease deaths**					
Maternal convictions	Non-violent	1.07 (0.97, 1.18)	.166	1.02 (0.93, 1.13)	.646
	Violent	1.04 (0.77, 1.39)	.810	0.94 (0.70, 1.27)	.699
Paternal convictions	Non-violent	1.07 (0.99, 1.14)	.074	1.03 (0.96, 1.11)	.404
	Violent	1.10 (0.98, 1.24)	.097	1.03 (0.91, 1.16)	.670
Infectious diseases					
Maternal convictions	Non-violent	1.22 (0.95, 1.57)	.127	1.09 (0.84, 1.41)	.524
	Violent	1.39 (0.72, 2.70)	.326	1.11 (0.57, 2.18)	.753
Paternal convictions	Non-violent	1.19 (0.99, 1.44)	.063	1.11 (0.92, 1.34)	.270
	Violent	**1.46 (1.10, 1.93)**	**.008**	1.24 (0.93, 1.66)	.139
Neoplasms					
Maternal convictions	Non-violent	0.98 (0.81, 1.18)	.802	0.97 (0.80, 1.17)	.718
	Violent	0.58 (0.29, 1.16)	.125	0.56 (0.28, 1.13)	.104
Paternal convictions	Non-violent	1.01 (0.88, 1.14)	.936	1.01 (0.88, 1.14)	.936
	Violent	1.06 (0.86, 1.31)	.584	1.08 (0.87, 1.34)	.483
Congenital malformations					
Maternal convictions	Non-violent	1.05 (0.86, 1.29)	.615	1.01 (0.82, 1.24)	.905
	Violent	1.17 (0.66, 2.07)	.596	1.09 (0.61, 1.93)	.780
Paternal convictions	Non-violent	1.11 (0.97, 1.28)	.128	1.08 (0.94, 1.25)	.269
	Violent	1.00 (0.78, 1.29)	.982	0.94 (0.73, 1.21)	.636
Other diseases					
Maternal convictions	Non-violent	1.12 (0.94, 1.34)	.191	1.06 (0.89, 1.27)	.495
	Violent	1.24 (0.76, 2.00)	.388	1.12 (0.69, 1.82)	.653
Paternal convictions	Non-violent	1.04 (0.92, 1.18)	.536	0.99 (0.87, 1.12)	.849
	Violent	1.07 (0.87, 1.32)	.516	0.95 (0.77, 1.18)	.632
**External-cause mortality**					
Maternal convictions	Non-violent	**1.62 (1.41, 1.87)**	**<.001**	**1.35 (1.17, 1.56)**	**<.001**
	Violent	**3.91 (3.03, 5.05)**	**<.001**	**2.71 (2.08, 3.52)**	**<.001**
Paternal convictions	Non-violent	**1.53 (1.37, 1.72)**	**<.001**	**1.40 (1.25, 1.57)**	**<.001**
	Violent	**2.52 (2.17, 2.93)**	**<.001**	**1.97 (1.68, 2.31)**	**<.001**
Accidents					
Maternal convictions	Non-violent	**1.57 (1.35, 1.82)**	**<.001**	**1.31 (1.13, 1.53)**	**<.001**
	Violent	**2.38 (1.69, 3.34)**	**<.001**	**1.67 (1.18, 2.35)**	**.004**
Paternal convictions	Non-violent	**1.58 (1.40, 1.77)**	**<.001**	**1.46 (1.30, 1.65)**	**<.001**
	Violent	**2.33 (1.98, 2.75)**	**<.001**	**1.94 (1.64, 2.30)**	**<.001**
Homicides					
Maternal convictions	Non-violent	1.46 (0.81, 2.64)	.212	1.22 (0.67, 2.24)	.511
	Violent	**27.89 (17.58, 44.24)**	**<.001**	**18.83 (11.34, 31.26)**	**<.001**
Paternal convictions	Non-violent	1.00 (0.63, 1.59)	.995	0.80 (0.50, 1.28)	.353

	Violent	**4.32 (2.73, 6.83)**	**<.001**	**2.06 (1.23, 3.43)**	**.006**
Other external-cause					
Maternal convictions	Non-violent	**3.85 (2.01, 7.38)**	**<.001**	**2.71 (1.38, 5.32)**	**.004**
	Violent	**4.53 (1.06, 19.33)**	**.041**	2.45 (0.56, 10.74)	.234
Paternal convictions	Non-violent	**2.03 (1.01, 4.08)**	**.047**	1.50 (0.74, 3.06)	.262
	Violent	**4.39 (1.96, 9.82)**	**<.001**	2.30 (0.99, 5.34)	.053

Note. Reference category for convictions: No convictions. Not enough cases for alcohol-related deaths and suicides in ages 1–9; these causes thus not analysed.

Model 1 controls for offspring's sex, mother's age at the child's birth and this in the second power, and child’s birth year. Model 2 controls for Model 1 and the co-parent’s criminality, parental education, and parental immigration status.

Disease deaths and external-cause mortality include all the respective sub-categories.

Table S4. Hazard ratios (HR) and 95% CIs from Cox regressions predicting offspring all-cause and cause-specific mortality in ages 10–19 by parental criminal convictions.

		Model 1		Model 2	
		HR (95%CI)	p	HR (95%CI)	p
**All-cause mortality**					
Maternal convictions	Non-violent	**1.38 (1.28, 1.48)**	**<.001**	**1.25 (1.16, 1.35)**	**<.001**
	Violent	**2.17 (1.82, 2.58)**	**<.001**	**1.78 (1.49, 2.13)**	**<.001**
Paternal convictions	Non-violent	**1.18 (1.11, 1.25)**	**<.001**	**1.12 (1.06, 1.19)**	**<.001**
	Violent	**1.73 (1.59, 1.89)**	**<.001**	**1.52 (1.39, 1.66)**	**<.001**
**Disease deaths**					
Maternal convictions	Non-violent	1.13 (0.99, 1.28)	.064	1.09 (0.96, 1.24)	.206
	Violent	**1.42 (1.02, 1.97)**	**.039**	1.29 (0.92, 1.80)	.136
Paternal convictions	Non-violent	0.98 (0.89, 1.07)	.654	0.95 (0.86, 1.04)	.251
	Violent	**1.21 (1.04, 1.40)**	**.011**	1.11 (0.95, 1.29)	.185
Infectious diseases					
Maternal convictions	Non-violent	**2.18 (1.50, 3.18)**	**<.001**	**2.00 (1.36, 2.95)**	**<.001**
	Violent	0.60 (0.08, 4.31)	.612	0.48 (0.07, 3.50)	.472
Paternal convictions	Non-violent	1.12 (0.79, 1.58)	.535	1.04 (0.73, 1.48)	.818
	Violent	**1.81 (1.12, 2.93)**	**.015**	1.56 (0.95, 2.58)	.080
Neoplasms					
Maternal convictions	Non-violent	0.85 (0.67, 1.07)	.175	0.88 (0.70, 1.12)	.307
	Violent	1.14 (0.63, 2.08)	.661	1.20 (0.66, 2.20)	.546
Paternal convictions	Non-violent	0.90 (0.77, 1.05)	.199	0.91 (0.78, 1.07)	.256
	Violent	0.85 (0.65, 1.12)	.247	0.87 (0.66, 1.15)	.319
Congenital malformations					
Maternal convictions	Non-violent	1.00 (0.68, 1.48)	.992	0.94 (0.63, 1.39)	.749
	Violent	1.34 (0.50, 3.62)	.559	1.13 (0.41, 3.08)	.814
Paternal convictions	Non-violent	0.89 (0.68, 1.18)	.424	0.86 (0.65, 1.14)	.301
	Violent	1.35 (0.90, 2.02)	.147	1.23 (0.81, 1.87)	.337
Other diseases					
Maternal convictions	Non-violent	**1.24 (1.03, 1.49)**	**.022**	1.15 (0.95, 1.38)	.150
	Violent	**1.80 (1.15, 2.80)**	**.010**	1.53 (0.97, 2.40)	.066
Paternal convictions	Non-violent	1.05 (0.91, 1.20)	.509	0.98 (0.85, 1.13)	.813
	Violent	**1.39 (1.13, 1.72)**	**.002**	1.19 (0.96, 1.48)	.108
**External-cause mortality**					
Maternal convictions	Non-violent	**1.57 (1.42, 1.73)**	**<.001**	**1.37 (1.24, 1.51)**	**<.001**
	Violent	**2.77 (2.25, 3.41)**	**<.001**	**2.12 (1.72, 2.62)**	**<.001**
Paternal convictions	Non-violent	**1.36 (1.26, 1.48)**	**<.001**	**1.28 (1.18, 1.39)**	**<.001**
	Violent	**2.21 (1.99, 2.46)**	**<.001**	**1.88 (1.68, 2.11)**	**<.001**
Alcohol-related deaths					
Maternal convictions	Non-violent	**1.99 (1.21, 3.27)**	**.007**	**1.73 (1.03, 2.89)**	**.037**
	Violent	2.70 (0.84, 8.64)	.094	1.81 (0.55, 5.94)	.326
Paternal convictions	Non-violent	1.01 (0.64, 1.60)	.970	0.89 (0.56, 1.42)	.631
	Violent	**2.25 (1.29, 3.92)**	**.004**	1.68 (0.93, 3.02)	.083
Accidents					
Maternal convictions	Non-violent	**1.55 (1.36, 1.76)**	**<.001**	**1.35 (1.19, 1.54)**	**<.001**

	Violent	**2.60 (1.97, 3.44)**	**<.001**	**2.02 (1.52, 2.68)**	**<.001**
Paternal convictions	Non-violent	**1.42 (1.29, 1.58)**	**<.001**	**1.33 (1.20, 1.47)**	**<.001**
	Violent	**2.14 (1.86, 2.46)**	**<.001**	**1.81 (1.56, 2.10)**	**<.001**
Suicides					
Maternal convictions	Non-violent	**1.38 (1.14, 1.66)**	**.001**	**1.26 (1.04, 1.53)**	**.018**
	Violent	**2.52 (1.70, 3.74)**	**<.001**	**2.12 (1.42, 3.17)**	**<.001**
Paternal convictions	Non-violent	**1.28 (1.10, 1.48)**	**.001**	**1.26 (1.08, 1.46)**	**.003**
	Violent	**1.89 (1.54, 2.32)**	**<.001**	**1.77 (1.42, 2.19)**	**<.001**
Homicides					
Maternal convictions	Non-violent	**2.23 (1.40, 3.55)**	**.001**	1.60 (0.99, 2.59)	.055
	Violent	**5.68 (2.46, 13.13)**	**<.001**	**3.01 (1.27, 7.14)**	**.012**
Paternal convictions	Non-violent	1.52 (0.98, 2.34)	.061	1.27 (0.81, 1.97)	.297
	Violent	**4.20 (2.56, 6.90)**	**<.001**	**2.69 (1.59, 4.57)**	**<.001**
Other external-cause					
Maternal convictions	Non-violent	**2.40 (1.59, 3.61)**	**<.001**	**1.85 (1.21, 2.82)**	**.005**
	Violent	**4.87 (2.24, 10.58)**	**<.001**	**2.81 (1.26, 6.25)**	**.012**
Paternal convictions	Non-violent	1.26 (0.84, 1.90)	.266	1.14 (0.75, 1.73)	.537
	Violent	**4.11 (2.66, 6.36)**	**<.001**	**3.15 (1.98, 5.02)**	**<.001**

Note. Reference category for convictions: No convictions. Model 1 controls for offspring's sex, mother's age at the child's birth and this in the second power, and child’s birth year. Model 2 controls for Model 1 and the co-parent’s criminality, parental education, and parental immigration status.
Disease deaths and external-cause mortality include all the respective sub-categories.

Table S5 shows results from Cox regressions predicting all-cause mortality in infancy, ages 1–9, and ages 10–19 by parental criminal convictions (mutually adjusted) and a set of other childhood risk factors. Missing information on parental education was coded as an additional category and included in the analyses. Being a boy was associated with a higher mortality risk relative to girls throughout childhood and adolescence, and this difference was most pronounced from age ten onwards. Mortality was also relatively higher in children of parents with lower education and with a mother with immigrant background, but the latter only until the 10th birthday.

Table S5. Hazard ratios (HR) and 95% CIs from Cox regressions predicting offspring all-cause mortality in different ages, by parental criminal convictions and some other childhood risk factors.

		Infancy		Ages 1–9		Ages 10–19	
		HR (95%CI)	p	HR (95%CI)	p	HR (95%CI)	p
**Parental criminality (ref. No convictions)**							
Maternal	Non-violent	1.14 (1.09, 1.19)	<.001	1.10 (1.02, 1.20)	.019	1.25 (1.16, 1.35)	<.001
	Violent	1.39 (1.23, 1.56)	<.001	1.47 (1.22, 1.79)	<.001	1.78 (1.49, 2.13)	<.001
Paternal	Non-violent	1.03 (1.00, 1.07)	.063	1.11 (1.05, 1.18)	<.001	1.12 (1.06, 1.19)	<.001
	Violent	1.11 (1.05, 1.17)	<.001	1.26 (1.15, 1.38)	<.001	1.52 (1.39, 1.66)	<.001
**Parental education (ref. High)**							
Mother's	Low	1.20 (1.14, 1.26)	<.001	1.37 (1.26, 1.50)	<.001	1.21 (1.11, 1.32)	<.001
	Middle	1.09 (1.05, 1.13)	<.001	1.12 (1.04, 1.19)	.001	1.10 (1.03, 1.18)	.004
	Missing	1.34 (1.15, 1.56)	<.001	2.27 (1.77, 2.92)	<.001	1.94 (1.37, 2.76)	<.001
Father's	Low	1.09 (1.04, 1.14)	.001	1.17 (1.08, 1.28)	<.001	1.13 (1.03, 1.22)	.006
	Middle	1.05 (1.01, 1.09)	.008	1.11 (1.03, 1.19)	.005	1.10 (1.03, 1.19)	.007
	Missing	0.82 (0.69, 0.96)	.013	1.12 (0.86, 1.45)	.401	1.15 (0.86, 1.54)	.341
**Parental immigration status (ref. Born in Sweden)**							
Mother immigrant		1.13 (1.07, 1.19)	<.001	1.16 (1.06, 1.27)	.001	1.04 (0.95, 1.15)	.381
Father immigrant		1.01 (0.96, 1.06)	.727	1.02 (0.93, 1.11)	.676	1.05 (0.95, 1.15)	.345
**Offspring's sex (ref. Girl)**		1.24 (1.21, 1.28)	<.001	1.26 (1.20, 1.33)	<.001	1.60 (1.52, 1.69)	<.001

Note. All models additionally control for mother's age at the child's birth and this in the second power, and child's birth year.

Supplementary Text 1. Testing the proportional hazards assumption.

We analysed mortality in infancy, ages 1–9, and ages 10–19 separately, and tested the proportional hazards assumption of Cox regression separately within all three time periods. We examined the correlations between the Schoenfeld residuals and analysis time for all predictors and covariates and visually compared Kaplan-Meier curves for the different levels of the main predictor, that is, maternal and paternal criminal convictions.

Due to the very large sample size, many of the correlations between analysis time and Schoenfeld residuals were significant, but the rhos were still negligible (all max. ± .06). In infancy, Schoenfeld residuals of maternal and paternal criminality correlated statistically significantly with time (albeit with very small effect sizes: rhos 0.02–0.04), and the Kaplan-Meier survival curves indicated that the effects of parental criminality were negligible during the first months and after which they started to show (Fig. S3). Due to computational limitations and the very small rhos, we nevertheless performed the main analysis in infancy without time-interaction terms. In the other time-periods, the Schoenfeld residuals of maternal and paternal criminality did not correlate with time significantly, and the Kaplan-Meier curves for different levels of parental criminality were relatively parallel (Figures S4 & S5).

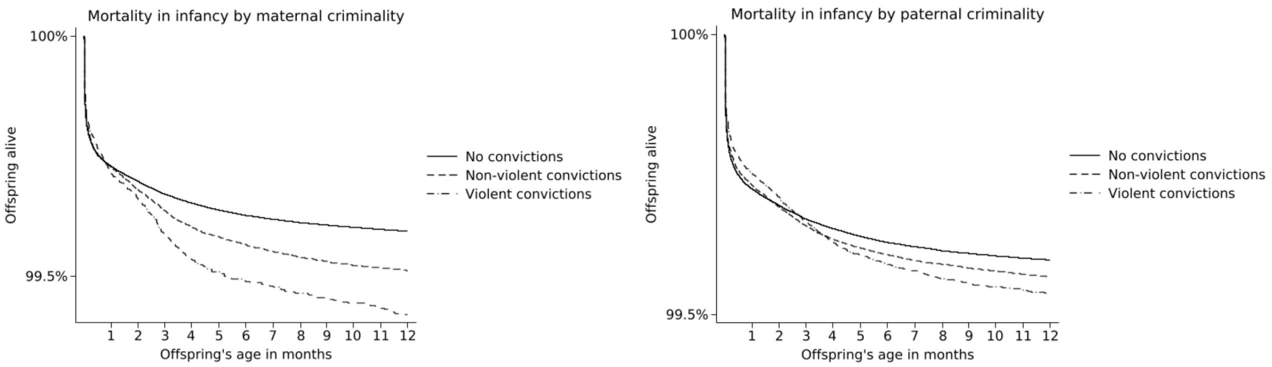

Figure S3. Observed (Kaplan-Meier) survival curves by parental criminal convictions in infancy.

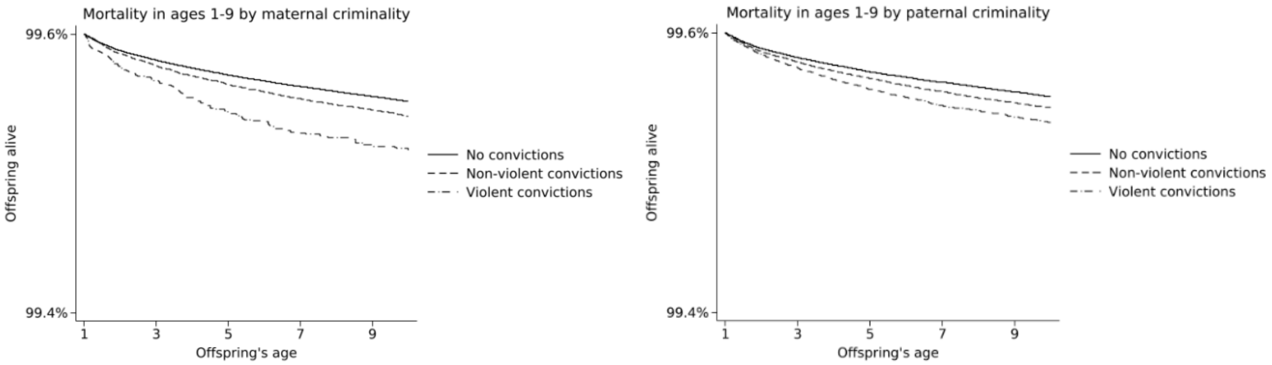

Figure S4. Observed (Kaplan-Meier) survival curves by parental criminal convictions in offspring ages 1–9.

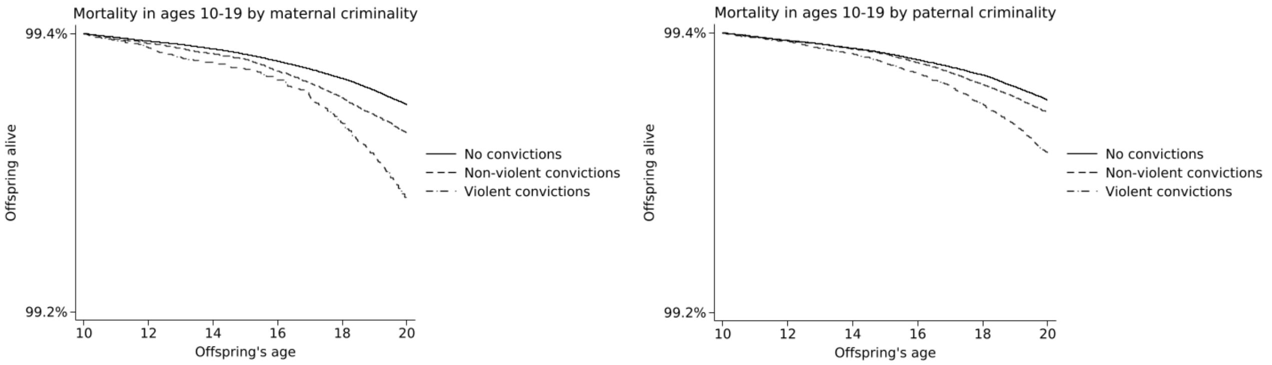

Figure S5. Observed (Kaplan-Meier) survival curves by parental criminal convictions in offspring ages 10–19.

For offspring sex in the analyses concerning 10–19-year-olds, the Schoenfeld residuals correlated somewhat more markedly with time (rho = 0.09), and from circa age 13 onwards, the mortality rate of boys started to deviate from that of girls (Fig. S6). However, since this deviation was very small in size and analyses concerning interactions between child sex and parental criminality showed no consistent statistically significant patterns (see main text), we decided to retain all analyses pooled for girls and boys to maintain statistical power when examining very rare causes of death. To state our reasoning more clearly, based on our analyses, we are confident that the differences in mortality between boys and girls that accelerate during puberty, are not to any meaningful extent driven by differing reactivity to parental criminal behaviour. Thus, analysing girls and boys pooled together is warranted within the scope of the current study.

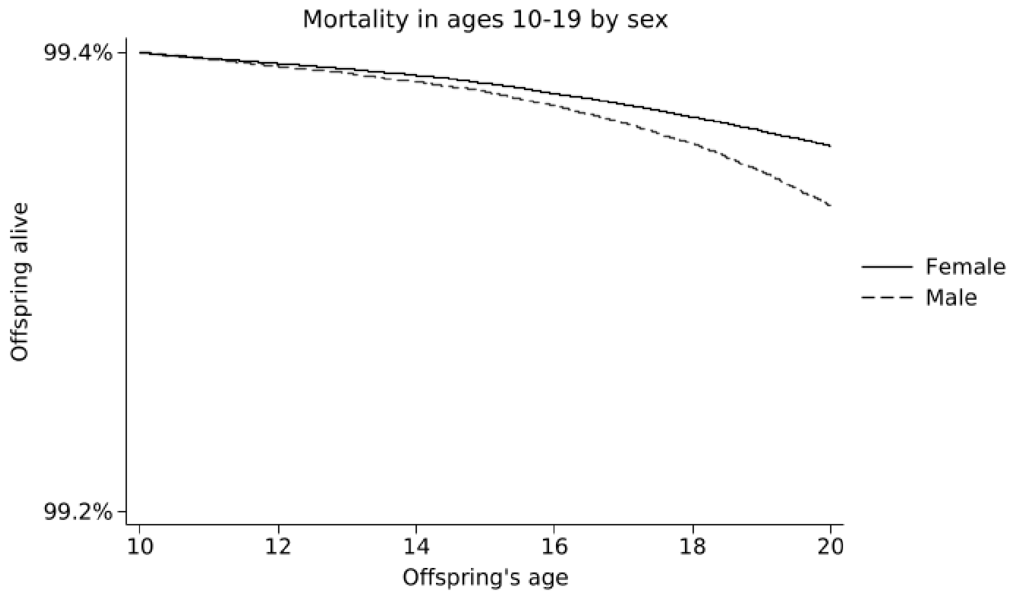

Figure S6. Observed (Kaplan-Meier) survival curves by offspring sex in offspring ages 10–19.

Table S6 present results from Cox regression with time-varying parental criminal conviction predicting all-cause mortality in infancy and ages 1–9 and 10–19. Time-varying parental criminal conviction predictors are assigned value “0” before the first conviction, and “1” or “2” thereafter, for parents without and with violent criminal convictions, respectively. The results for these models are largely similar to the main results, meaning that having a parent or parents with either non-violent or violent criminal convictions is associated with a heightened mortality risk for the offspring throughout the childhood and adolescence. The timing of parental convictions bears little meaning on the strength of these associations.

Table S6. Hazard ratios (HR) and 95% CIs from Cox regressions predicting offspring all-cause mortality by parental convictions in a time-varying model.

		Model 1		Model 2	
		HR (95%CI)	p	HR (95%CI)	p
Mortality in infancy					
Maternal convictions	Non-violent	**1.19 (1.12, 1.27)**	**.000**	**1.14 (1.08, 1.21)**	**.000**
	Violent	**1.56 (1.34, 1.82)**	**.000**	**1.42 (1.22, 1.65)**	**.000**
Paternal convictions	Non-violent	**1.07 (1.03, 1.11)**	**.000**	**1.04 (1.00, 1.08)**	**.035**
	Violent	**1.19 (1.13, 1.27)**	**.000**	**1.10 (1.04, 1.17)**	**.002**
Mortality in ages 1–9					
Maternal convictions	Non-violent	**1.24 (1.12, 1.36)**	**.000**	**1.12 (1.02, 1.24)**	**.020**
	Violent	**1.47 (1.14, 1.90)**	**.003**	1.20 (0.93, 1.55)	.159
Paternal convictions	Non-violent	**1.13 (1.06, 1.21)**	**.000**	**1.08 (1.01, 1.15)**	**.020**
	Violent	**1.40 (1.27, 1.54)**	**.000**	**1.25 (1.13, 1.38)**	**.000**
Mortality in ages 10–19					
Maternal convictions	Non-violent	**1.42 (1.31, 1.53)**	**.000**	**1.26 (1.16, 1.37)**	**.000**
	Violent	**2.27 (1.89, 2.73)**	**.000**	**1.82 (1.51, 2.19)**	**.000**
Paternal convictions	Non-violent	**1.22 (1.14, 1.29)**	**.000**	**1.15 (1.08, 1.23)**	**.000**
	Violent	**1.77 (1.63, 1.93)**	**.000**	**1.54 (1.41, 1.68)**	**.000**

Model 1 controls for offspring's sex, mother's age at the child's birth and this in the second power, and child’s birth year. Model 2 controls for Model 1 and the co-parent’s criminality, parental education, and parental immigration status.

Table S7 reports the associations between parental criminality and child all-cause mortality, excluding children who died of homicide. By these analyses, we wanted to test how much of the effect between parental criminality and offspring mortality is driven by possible overlap of the predictor and response variable, i.e., the possibility that the parent has been convicted for the filicide of the focal child. Of the 251 children died of homicide during the follow-up, around 40% had a parent with a violent crime conviction. Thus, excluding all children who died of homicide provides a conservative, minimum estimate for the association between parental criminality and child mortality without the possible filicide-cases interfering the analysis.

Table S7. Hazard ratios (HR) and 95% CIs from Cox regressions predicting offspring all-cause mortality by parental criminal convictions, excluding homicidal deaths.

		Model 1		Model 2	
		HR (95%CI)	p	HR (95%CI)	p
**Mortality in infancy**					
Maternal convictions	Non-violent	**1.19 (1.14, 1.24)**	**<.001**	**1.18 (1.12, 1.24)**	**<.001**
	Violent	**1.47 (1.31, 1.66)**	**<.001**	**1.31 (1.14, 1.51)**	**<.001**
Paternal convictions	Non-violent	**1.07 (1.03, 1.11)**	**<.001**	**1.04 (1.01, 1.08)**	**.021**
	Violent	**1.20 (1.14, 1.27)**	**<.001**	**1.10 (1.03, 1.17)**	**.002**
**Mortality in ages 1–9**					
Maternal convictions	Non-violent	**1.20 (1.11, 1.30)**	**<.001**	**1.11 (1.02, 1.22)**	**.019**
	Violent	**1.37 (1.10, 1.70)**	**.004**	1.21 (0.95, 1.54)	.130
Paternal convictions	Non-violent	**1.17 (1.11, 1.24)**	**<.001**	**1.09 (1.02, 1.17)**	**.008**
	Violent	**1.38 (1.26, 1.51)**	**<.001**	**1.19 (1.07, 1.33)**	**.001**
**Mortality in ages 10–19**					
Maternal convictions	Non-violent	**1.36 (1.26, 1.47)**	**<.001**	**1.21 (1.11, 1.31)**	**<.001**
	Violent	**2.11 (1.77, 2.52)**	**<.001**	**1.70 (1.40, 2.07)**	**<.001**
Paternal convictions	Non-violent	**1.17 (1.11, 1.25)**	**<.001**	**1.10 (1.03, 1.17)**	**.003**
	Violent	**1.69 (1.55, 1.85)**	**<.001**	**1.43 (1.30, 1.57)**	**<.001**

Note. Reference category for convictions: No convictions. Model 1 controls for offspring's sex, mother's age at the child's birth and this in the second power, and child’s birth year. Model 2 controls for Model 1 and the co-parent’s criminality, parental education, and parental immigration status.

This sensitivity analysis shows that, in all ages, the associations between non-violent parental criminality and child all-cause mortality are little affected by excluding homicidal deaths, indicating that these associations are robust to excluding a small number of cases. The associations between parental violent criminality and offspring mortality, however, were more markedly attenuated when excluding homicidal deaths, with the largest reductions in childhood and adolescence, and less pronounced changes in infancy. The smaller changes in the point estimates in infancy reflect the fact that homicidal deaths accounted for a smaller proportion of all deaths in infancy than in the other two age periods. Therefore, even with the very large effect sizes for the association between parental violent criminality and offspring homicidal mortality in infancy (main text, Table 2), excluding these cases only marginally reduced the overall association for all-cause mortality.

In the fully adjusted models, the hazard ratios between maternal or paternal violent criminality and offspring all-cause mortality were attenuated by 18 % and 6 %, respectively, in ages 1–9 and by 4 % and 6 % in ages 10–19. The association between maternal violent criminality and offspring all-cause mortality in ages 1–9 was no longer statistically significant when excluding the homicidal deaths.

Figures S7–S8 show the frequencies of maternal and paternal criminal convictions in relation to focal child’s date of birth and, for the subsample of deceased children, to date of death. Only one event per child–mother or child–father pair is shown, that is, the time elapsed between the parent’s first criminal conviction and childbirth/death.

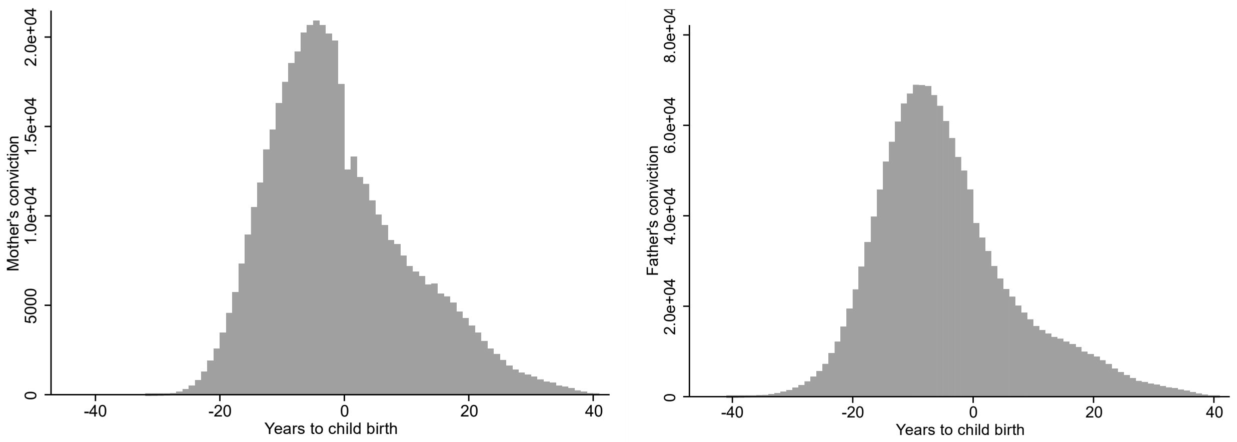

Figure S7. Histograms of mother's (on the left) and father's (on the right) first criminal conviction in relation to childbirth. Note the different scales on the y-axis.

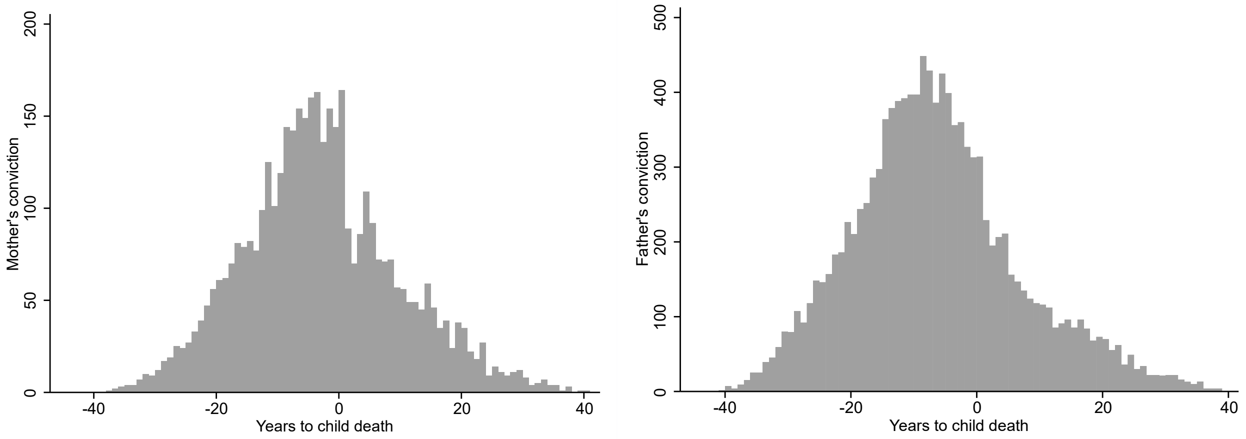

Figure S8. Histograms of mother's (on the left) and father's (on the right) first criminal conviction in relation to child death in the subsample of deceased children. Note the different scales on the y-axis.

Parental first criminal convictions were concentrated around 10–15 years before the focal child's birth (Fig. S7), which matches the fact that criminal behaviour most commonly starts in adolescence (Jolliffe et al., 2017). In mothers, a childbirth was associated with a rather sharp decline in commencing criminal behaviour whereas no such effect was observed in fathers. The relation between parental convictions and focal children's death follows the same patterns (Fig. S8), mostly due to most of the deaths occurring by the first birthday. As is evident from the histograms, a significant proportion of first parental criminal convictions in registers occur only after the focal child's death. Based on register data, it is impossible to say whether the parent has behaved antisocially even before the first conviction. In any case, the histograms do not show a pattern that would be indicative of reverse causality, i.e., that a child's death would increase the likelihood of a parental criminal conviction. In summary, the patterns between timing of parents' convictions and focal children's childhood seem to warrant the approach that parental criminal behaviour can be treated as a risk marker for instable childhood conditions that are associated with a higher mortality risk.
